# Longitudinal two-photon calcium imaging with ultra-large cranial window for head-fixed mice

**DOI:** 10.1016/j.xpro.2022.101343

**Published:** 2022-04-22

**Authors:** Ryoma Hattori, Takaki Komiyama

**Affiliations:** 1 Neurobiology Section, Center for Neural Circuits and Behavior, Department of Neurosciences, and Halıcıoğlu Data Science Institute, University of California, San Diego, La Jolla, CA 92093, USA

**Keywords:** Microscopy, Model Organisms, Neuroscience

## Abstract

Neural activity is heterogeneous across different cortical areas and can change during learning. Here, we describe a protocol for longitudinal *in vivo* two-photon calcium imaging with an ultra-large cranial window that exposes most of the dorsal cortex in head-fixed mice. The large cranial window allows optical access to any dorsal cortical areas in individual mice. This protocol enables longitudinal tracking of neural activity from various cortical areas at cellular resolution to understand the cortical computations during behavioral tasks.

For complete details on the use and execution of this protocol, please refer to Hattori et al. (2019), and Hattori and Komiyama, 2022a.

## Highlights

• Ultra-large craniotomy for *in vivo* two-photon imaging
• Ultra-large cranial window allows optical access to the entire dorsal cortex
• Calcium imaging data can be collected from many cortical areas from individual mice
• Longitudinal calcium imaging tracks neural activity changes across days

## Graphical abstract

**Figure:**
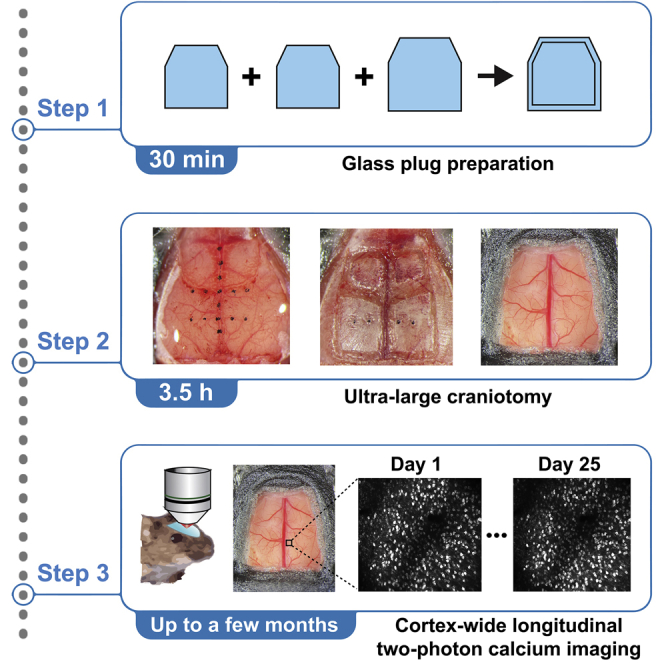

## Before you begin

Two-photon calcium imaging is a powerful approach to measure neuronal activity at high spatial resolution (Grienberger and Konnerth, 2012; Svoboda and Yasuda, 2006). The technique relies on fluorescent calcium indicators that alter their fluorescence in response to changes in the intracellular calcium concentration. Calcium ions flow into the intracellular compartment through voltage-gated channels in response to neuronal spiking activity. The subcellular resolution of two-photon microscopy and the fluorescent calcium indicators allow the indirect measurement of spiking activity. Furthermore, the activity of the same neurons can be tracked reliably for many weeks and months, which is challenging with electrode recordings.

In this paper, we describe a protocol for longitudinal *in vivo* two-photon calcium imaging through an ultra large glass window from behaving mice. The large glass window preparation allows optical access to most dorsal cortical areas in individual mice (Hattori and Komiyama, 2022a; Hattori et al., 2019). The protocol was inspired by an earlier study that described a similar approach (Kim et al., 2016). We describe the step-by-step procedures of the surgery and the imaging experiments.

### Institutional permissions

All animal experiments were performed in accordance with the institutional guidelines and were approved by the Institutional Animal Care and Use Committee (IACUC) at the University of California, San Diego. Before performing the animal experiments in the protocol, the protocol needs to be approved at respective institutions.

### Preparation of animals

**Timing: ∼3 months**

1. Cross CaMKIIα-tTA transgenic mice (Mayford et al., 1996) with tetO-GCaMP6s transgenic mice (Wekselblatt et al., 2016). The double transgenic mice express the genetically encoded calcium indicator, GCaMP6s (Chen et al., 2013), in most cortical excitatory neurons. Select the genetic backgrounds of animals according to the specific cell types of your interest.

***Note:*** Multiple transgenic mouse lines for calcium imaging are available. The mouse line should be selected based on the type of the expressed calcium indicator, the specificity and strength of its expression, and the health of the expressing neurons. Some transgenic mice with GCaMP expression are reported to show aberrant cortical activity (Steinmetz et al., 2017). The study did not observe such aberrant cortical activity in CaMKIIα-tTA::tetO-GCaMP6s double transgenic mice.

***Note:*** When longitudinal calcium imaging is necessary, we recommend the use of transgenic mice that endogenously express a calcium indicator instead of virally expressing an indicator. Long-term viral expressions of calcium indicators can result in overexpression, leading to abnormal neuronal activity and cell death. Long-term stable expression can be achieved by viral expression by adjusting viral titers. If viral expression is necessary, confirm that the cellular morphology and the fluorescence patterns are stable for the entire duration of the planned imaging period.

### Preparation of glass plugs for cranial windows

**Timing: 30 min**

**Figure 1:**
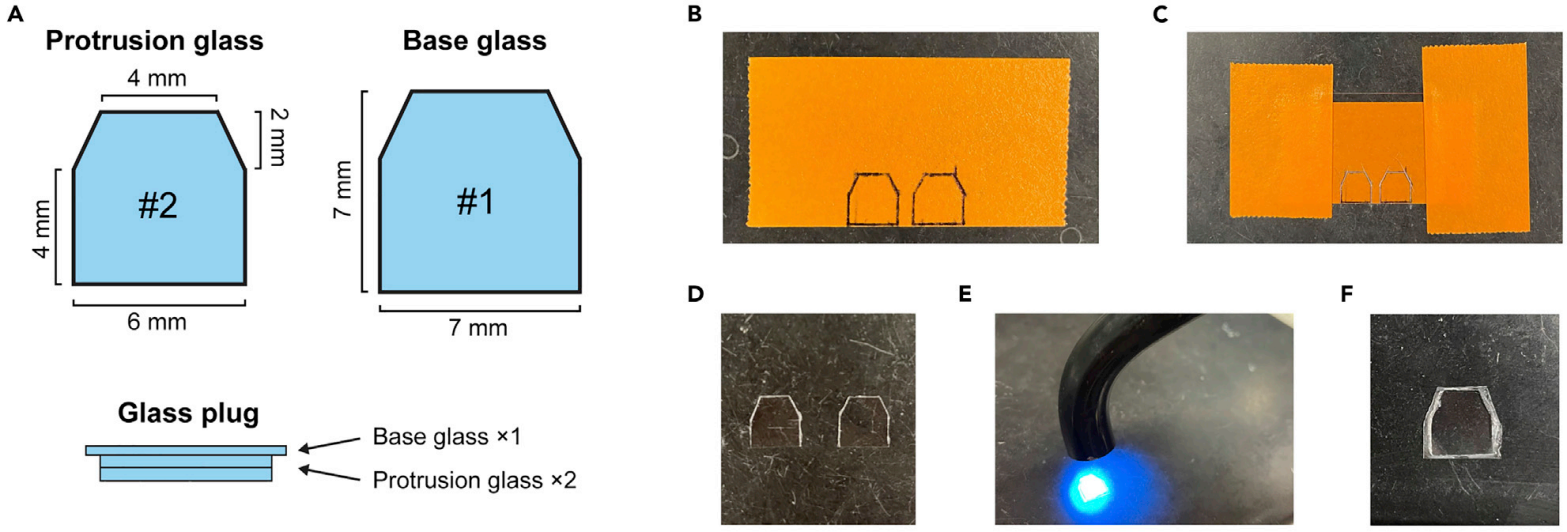
Preparation of glass plugs for large cranial windows (A) Two pieces of the protrusion glass and one piece of the base glass are made using #2 and #1 coverslips, respectively. They are glued together to make a glass plug. (B) Drawings of the shape on a piece of tape. (C) A coverslip is taped on the drawing and is scraped with a diamond scribe. (D) Two pieces of the protrusion glass cut out from the coverslip. (E) Glass pieces are glued by UV glue. (F) Assembled glass plug (1 base glass + 2 protrusion glasses).

This section describes the steps to make a glass plug that will be implanted on a mouse brain as the cranial window (Figure 1A).

2. Make the protruding part of a glass plug (“protrusion glass”).
  a. Draw the shape of a window on a piece of tape using a pen. We used a hexagonal window (Figure 1B).
  b. Place a #2 coverslip (∼220 μm thickness) over the drawings and fix it with tapes.
  c. Scrape the surface of the coverslip along the drawing using a diamond scribe and a ruler (Figure 1C).
  d. Detach the coverslip from the tape and carefully crack the glass with forceps along the scribed lines (Figure 1D).

***Note:*** Some variability in the cut-out window size is inevitable. We recommend making many pieces and select those that fit the desired size in step 4.

3. Make the base of a glass plug (“base glass”). Repeat step 2 with a #1 coverslip (∼150 μm thickness) and a slightly larger drawing (increase the size by ∼1 mm).
4. Make a glass plug by stacking 2 protrusion glasses and 1 base glass (Figure 1A).
  a. Clean the glasses using Kimwipe soaked with 70% ethanol. Make sure that there is no dust on the glasses before proceeding to the next step.
  b. Drop a small amount of UV glue (< 1 μL) on the center of the base glass using a pipette (10 μL or 20 μL pipette tip).
    ***Note:*** Make sure that the droplet of the UV glue does not contain any air bubbles. Air bubbles can be removed by centrifuging the glue in a tube (e.g., 3,000 × *g* for 5 s).
  c. Carefully place a protrusion glass on the base glass, avoiding air bubbles.
  d. Glue them by illuminating with UV light (Figure 1E). Adjust the illumination time according to the glue type and UV light intensity, typically 5–10 s.
  e. Repeat steps b-d to stack another protrusion glass.
  f. Remove extra glue on the base glass using fine forceps.
    ***Note:*** Sufficient height difference between the protrusion part and the base part is necessary to add enough pressure on the cortical areas, which minimizes motion artifacts during imaging from behaving animals. It also decreases the chance of getting bone growth from the edges of cranial window. If too much glue remains on the base glass, the protruding part of the glass plug will not have enough height. The double stacking of #2 coverslip has ∼440 μm height, which is sufficient to span the skull thickness and add gentle pressure on the cortex.

5. Keep the glass plug in 70% ethanol until surgery.

### Preparation of surgical tools

**Timing: 10 min**

This section describes the preparation of surgical tools.

6. Sterilize the surgery table and the stereotaxic instrument with 70% ethanol.
7. Sterilize forceps, scissors, scalpel, double-ended carver, head bar, and drill bits using a hot bead sterilizer.
8. Cut gelatin sponge into small pieces (∼1 mm square) and soak them in saline.

## Key resources table

**Table:** 

REAGENT or RESOURCE	SOURCE	IDENTIFIER
**Chemicals, peptides, and recombinant proteins**		

Isoflurane	VetOne	Cat#502017
Saline (0.9% sodium chloride)	ICU Medical	NDC 0990-7983-09
Vaseline Healing Jelly Original	Vaseline	N/A
Betadine (Solimo First Aid Antiseptic, 10%)	Solimo	N/A
Krazy glue (Cyanoacrylate glue)	Krazy Glue	Cat#KG585
Dental acrylic cement (Contemporary Ortho-Jet™ Powder and Liquid)	Lang Dental	N/A
3M Vetbond	3M	Cat#1469SB
Norland optical adhesive 61 (UV glue)	Norland Products	Cat#6101
Buprenorphine Hydrochloride (0.3 mg/mL)	Par Pharmaceutical	NDC 42023-179-05
Baytril (100 mg/mL)	Bayer	NADA 141-068
Dexamethasone (2 mg/mL)	VetOne	Cat#501012

**Experimental models: Organisms/strains**		

Mouse: CaMKIIa-tTA, B6;CBA-Tg(Camk2a-tTA)1Mmay/J (> P60, both females and males)	The Jackson Laboratory	RRID: IMSR_JAX: 003010
Mouse: tetO-GCaMP6s, B6;DBA-Tg(tetO-GCaMP6s)2Niell/J (> P60, both females and males)	The Jackson Laboratory	RRID: IMSR_JAX: 024742

**Software and algorithms**		

MATLAB	MathWorks	RRID: SCR_001622https://www.mathworks.com/products/matlab.html
ScanImage	Vidrio Technologies, LLC	RRID:SCR_014307https://vidriotechnologies.com/scanimage
PatchWarp	(Hattori and Komiyama, 2022b)	https://doi.org/10.5281/zenodo.5232757https://github.com/ryhattori/PatchWarp

**Other**		

UV Curing LED System, 365 nm	Thorlabs	Cat# CS20K2
#1 cover glass	Fisher Scientific	Cat#12-542-B
#2 cover glass	Fisher Scientific	Cat#12-540-B
Diamond scribe	Fiber Instrument Sales	Cat#F090C
Mini Centrifuge	VWR	Cat# 76269-064
Stereotaxic instrument	KOPF	Cat# Model 1900
Leica M125C stereomicroscope	Leica Microsystems	Cat#M125 C
Schott KL 1500 LED (Fiber optic light source with gooseneck)	SCHOTT	N/A
Mouse gas anesthesia head holder (nose cone and bite bar)	KOPF	Cat#1923-B
Stainless steel head bar	Custom-made from eMachineShop	N/A
Dental drill handpiece	Midwest Tradition	N/A
Drill bit (FG 1/4)	Henry Schein	Cat#1007205
Scalpel (#3)	Fine Science Tools	Cat#10003-12
Fine scissors	Fine Science Tools	Cat#14060-11
Forceps	Fine Science Tools	Cat#11223-20
Double-end carver	Henry Schein	Cat#101-0333
Compressed air duster	Office Depot	Cat#337994
Surgifoam (gelatin sponge)	Ethicon	Cat#1975
Servo-controlled heating pad	Harvard Apparatus	Cat#50-7220F
Hot bead sterilizer	Fine Science Tools	Cat#18000-45
Isoflurane vaporizer and anesthesia system	Vetequip	Cat#901806
Induction chamber for gas anesthesia	Vetequip	Cat#941444
B-SCOPE (Multiphoton Imaging Microscope)	Thorlabs	Cat#B-SCOPE
Digital Angle Gauge	Wixey	Cat#WR300-Type 2

## Step-by-step method details

### Preparation for craniotomy

**Timing: 30 min**

This section describes the surgical steps to prepare the mouse skull for craniotomy.

1. Anesthetize the adult mouse (> 2 months old) in a chamber filled with 3% isoflurane in oxygen.
2. Clamp the nose of the mouse on the stereotaxic instrument (Figure 2A). Isoflurane (1%–1.5% in oxygen) is provided from the nose clamp during the surgery. The body temperature is maintained by a servo-controlled heating pad.

**Figure 2:**
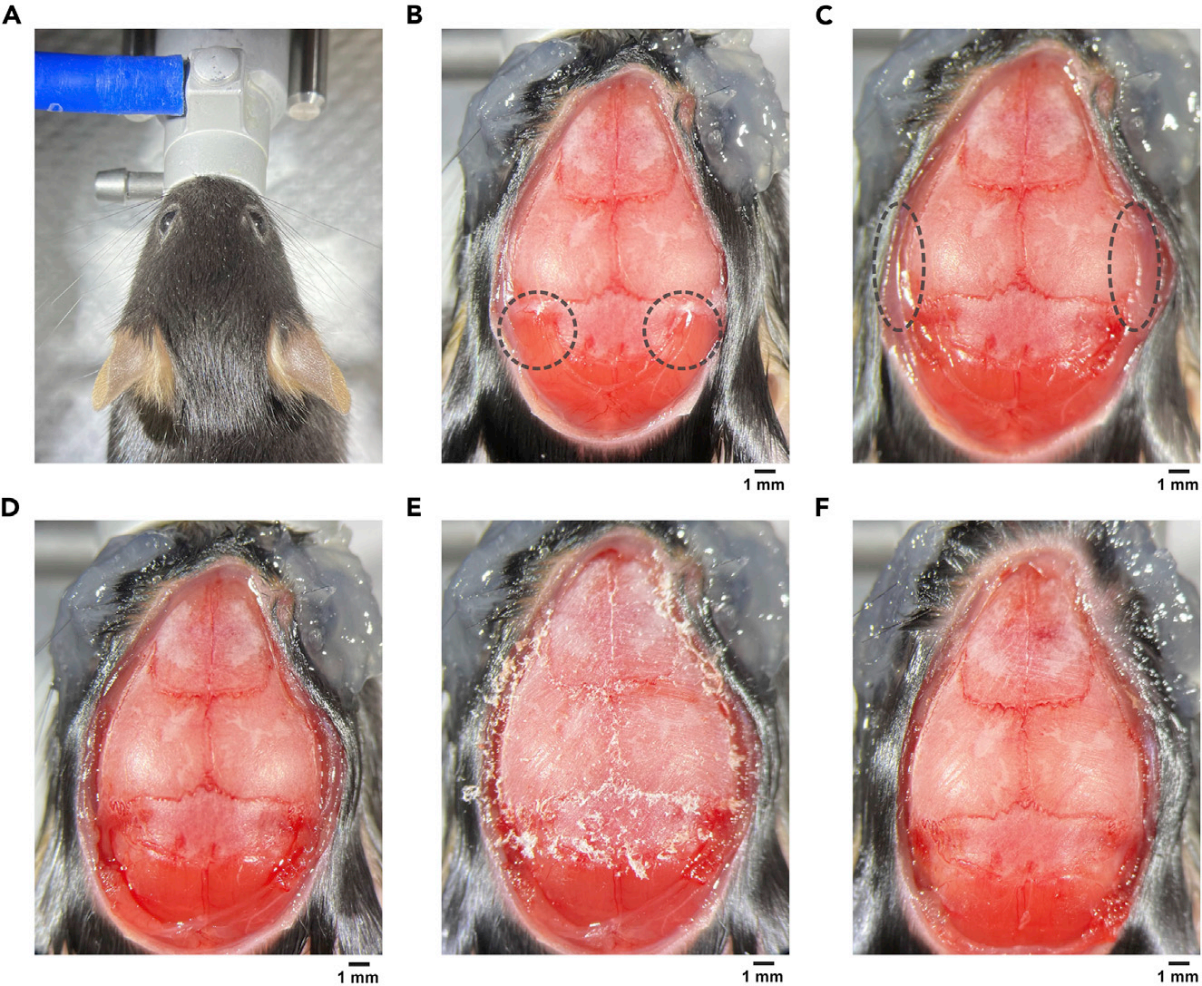
Preparation for craniotomy (A) A mouse is fixed by a nose clamp. (B) After opening the skin. The muscles above the cerebellum skull (dashed circles) will be detached next. (C) The muscles above the cerebellum have been detached. The lateral muscles near the auditory cortex (dashed circles) will be detached next. (D) After detaching both muscles. (E) Connective tissue on the skull surface was scraped off using a scalpel. (F) After cleaning the skull surface with 70% ethanol.
3. Inject baytril (10 mg/kg) and dexamethasone (2 mg/kg) subcutaneously to prevent possible infection and inflammation.
4. Disinfect the head skin with 70% ethanol and betadine.
5. Cover eyes with Vaseline to keep eyes from drying.
6. Cut out and remove the skin above the skull with scissors. Expose the dorsal skull above the olfactory bulb, cerebral cortex, and cerebellum.
7. Clean the skull surface using Kimwipe soaked with 70% ethanol.
8. Detach muscles on the lateral edges of cerebellum to increase skull surface area (Figures 2B and 2C). Leave the detached muscles posterior to the skull.
9. Detach lateral muscles near auditory cortex to increase skull surface area (Figures 2C and 2D).
  a. Leave the detached muscles at the original place.
  b. Krazy glue and dental cement will be placed in the ‘pocket’ between the lateral muscle and the skull in steps 33 and 34.

**CRITICAL:** These steps of increasing skull surface area are critical to secure a head bar on the skull in later steps because a large portion of the dorsal skull will be removed for the ultra large cranial window.

10. Scrape off the connective tissue on the skull surface using a scalpel (Figure 2E).

***Note:*** Make sure to completely remove the connective tissue. Remaining tissue loosens the head bar attachment in later steps.

11. Clean the skull surface using Kimwipe soaked with 70% ethanol (Figure 2F).

### Marking the stereotaxic coordinates

**Timing: 40 min**

This section describes the steps to mark the stereotaxic coordinates on the mouse skull. A photo with the marks and the cortical vasculature patterns will be used to find the target cortical areas during two-photon imaging.

**Figure 3:**
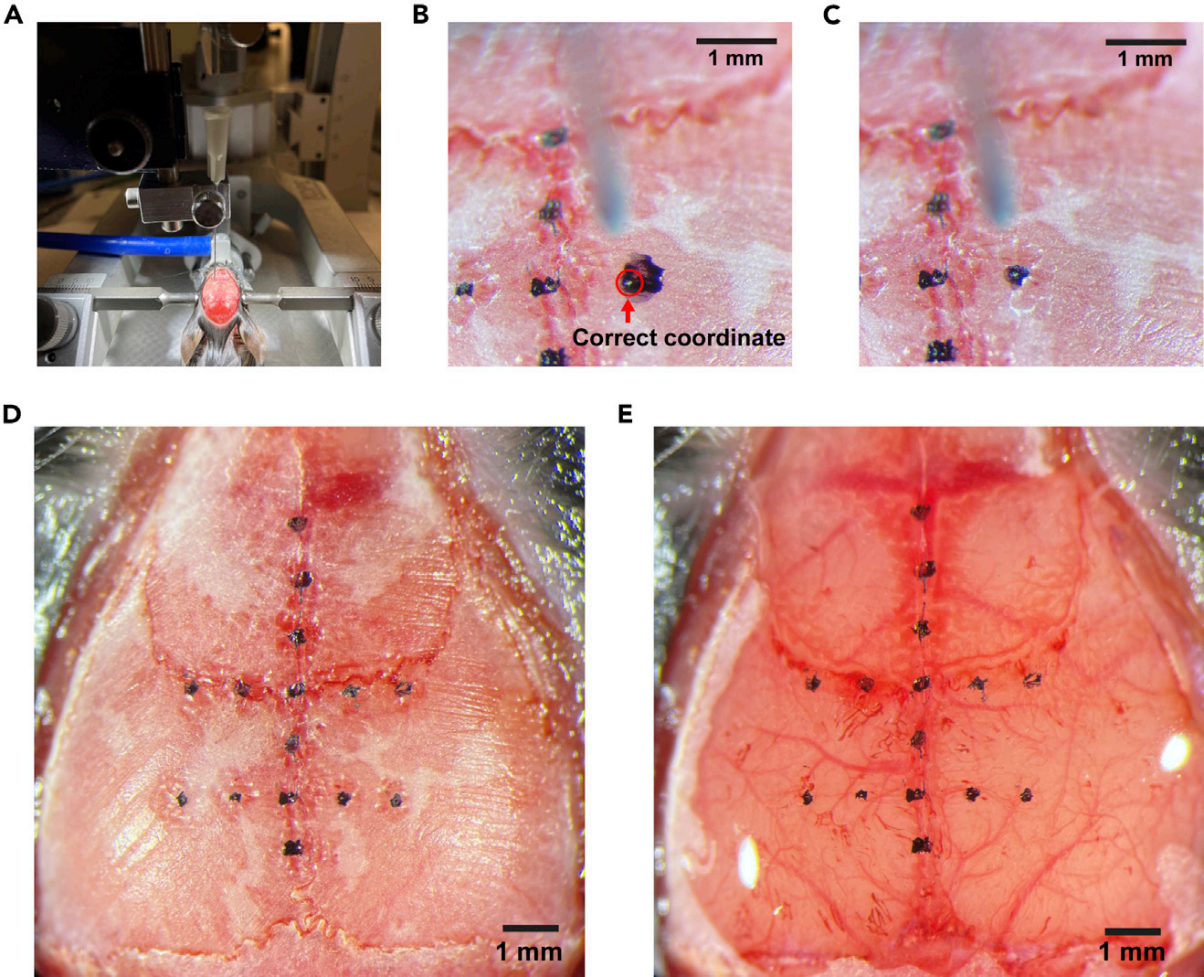
Marking the stereotaxic coordinates (A) A needle on a manipulator will be used to measure the coordinate from the bregma. (B) A rough marking with a permanent pen. The white dot on the ink (highlighted by a red circle) was marked by sticking the needle to confirm the accurate coordinate. (C) Extra ink was removed by a drill to make sure that the correct coordinate is at the center of the ink. (D) After marking all coordinates. Note that the midline sinus and the midline suture line are slightly misaligned in this mouse around the posterior cortex. The ML coordinates for the posterior areas started from the midline sinus instead of the suture line for this animal. (E) The skull was made semi-transparent by saline application, visualizing the vasculature patterns on the dorsal cortex. A photo of this image will be used as a reference to find target imaging areas under a two-photon microscope.
  b. Equalize the heights between the bregma and the lambda and the heights between ±2 mm lateral from the bregma.

12. Level the skull using the stereotaxic instrument.
  a. Use a needle fixed on a motorized micromanipulator to measure the height at each coordinate (Figure 3A).

13. Mark the following coordinates on the skull using a water-resistant permanent marker. [Anteroposterior (AP) coordinate from bregma, Mediolateral (ML) coordinate from bregma] = [0 mm, 0 mm], [±1.0 mm, 0 mm], [±2.0 mm, 0 mm], [±3.0 mm, 0 mm], [0 mm, ±1.0 mm], [0 mm, ±2.0 mm], [-2.0 mm, ±1.0 mm], [-2.0 mm, ±2.0 mm].
  ***Note:*** These marking coordinates can be changed based on the size of craniotomy and target cortical areas. We recommend including at least 2 lines that span along ML axis so that we can interpolate the missing coordinates within the cranial window.
  a. Find each coordinate using the needle.
  b. Mark the coordinate using the marker (Figure 3B).
  c. Confirm the coordinate by sticking the needle down to the ink.
  d. Remove extra ink by a drill (Figure 3C). The needle mark from step c needs to be at the center of the ink mark. Although the manually marked coordinates at step b are not very accurate, this extra drilling step improves the accuracies.
    ***Note:*** Midline sinus is occasionally misaligned with the midline suture on the skull (Figures 3D and 3E). Confirm the alignment by making the skull semi-transparent by placing a drop of saline and wait for ∼30 s. If large misalignment is found, the ML coordinates for the marks should begin from the midline sinus under the skull instead of the suture line at each AP coordinate.

14. Cover the dorsal skull with saline and wait for at least 30 s to make the skull semi-transparent.
15. Take a photo of the semi-transparent skull with the marked coordinates and the vasculatures on the cortex (Figure 3E).

***Note:*** Older mice occasionally have thicker skulls, and their skulls may not become transparent enough to visualize the cortical vasculatures. If the transparency is not sufficient, thin the skull by drilling until you get sufficient transparency.

### Ultra large craniotomy and window implantation

**Timing: 1.5 h**

This section describes the steps to make a large cranial window that exposes most of the dorsal cortex.

16. Wash the glass plug with saline.
17. Place the glass plug on the skull to check the necessary craniotomy size for the window implantation.

***Note:*** The craniotomy size needs to be larger than the protrusion glass.

**Figure 4:**
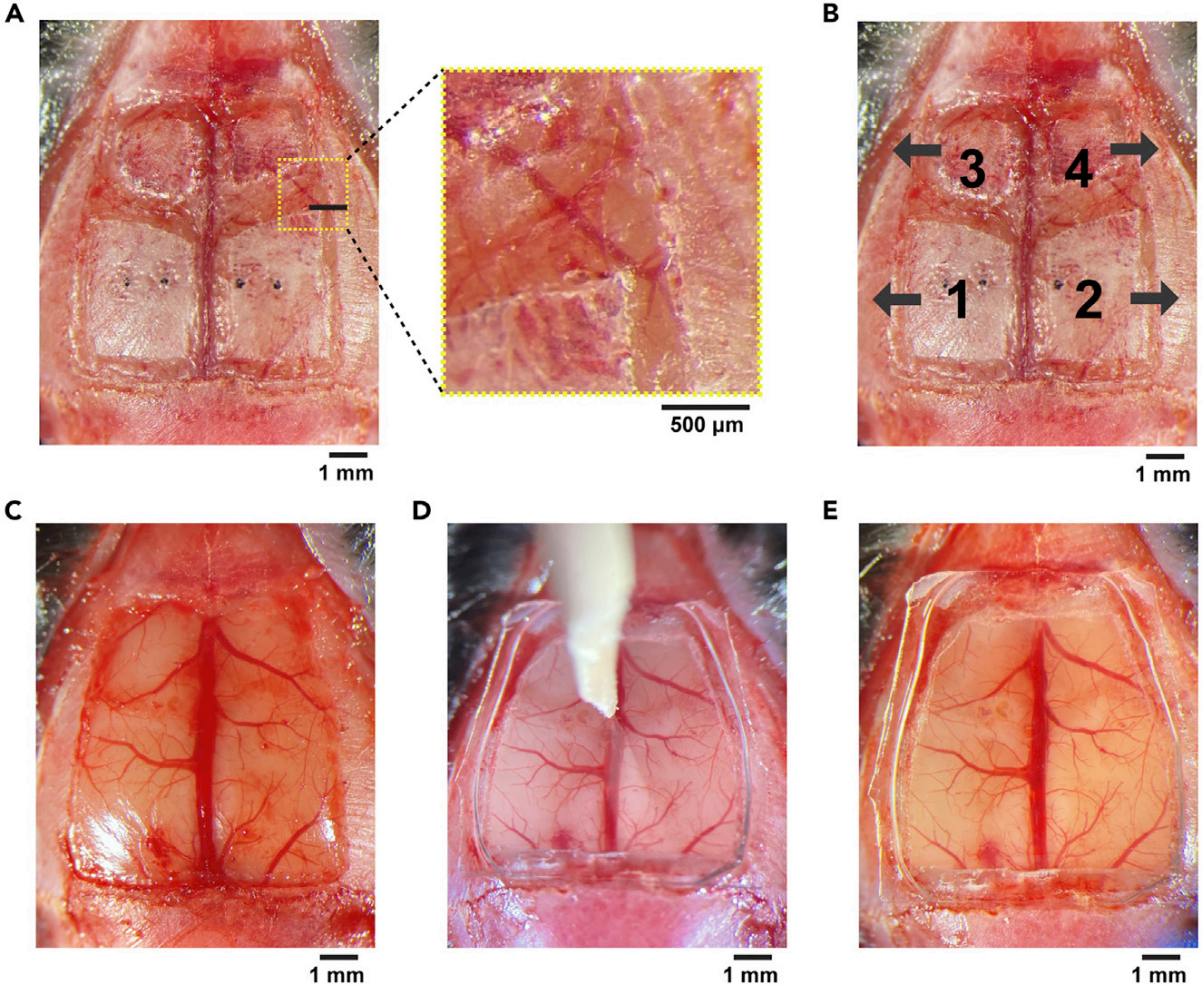
Ultra large craniotomy and window implantation (A) The dorsal skull is thinned along the edges of the cranial window and the suture lines. The skull needs to be thinned until the underlying vasculature pattern becomes clearly visible as shown in the zoomed image. (B) The split bones will be removed in the ascending order from #1 to #4. Grab the bone with forceps and slowly slide it toward the arrow direction on the image. (C) After removal of the bones. Dura is kept intact. (D) A glass plug is pressed on the cortex, and the position is fixed with 3M Vetbond and Krazy glue. (E) After the glass implantation.

***Note:*** Do not penetrate the skull with a drill. Thin the skull as much as possible without exposing the brain. To stop bleeding, place a saline-soaked gelatin sponge on the bleeding site.

18. Take off the glass plug and thin the skull along the edges of the intended cranial window using a drill (Figure 4A).
  a. No need to mark the outline of the window on the skull, but frequently place the glass plug on the skull during skull thinning to make sure that the size of your craniotomy is sufficient for the glass window.
  b. Use a compressed air duster to clear the skull debris.

19. Thin the skull along the midline and coronal suture lines (Figure 4A).

**CRITICAL:** The skull along the suture lines are especially sticky to the dura. Dura will be damaged if you skip this step when the skull is removed in later step. Skull thinning followed by saline exposure makes it easier to detach the skull from the dura and the sinus.

20. Cover the dorsal skull with saline and wait for at least 1 min.

***Note:*** This step makes the skull less sticky to the dura.

21. Remove the skull in the order of left parietal bone, right parietal bone, left frontal bone, and right frontal bone by the following procedure (Figures 4B and 4C). Keep saline on the skull throughout these steps.
  a. Carefully and slowly lift the bone from its edge using the fine forceps.
  b. Detach the bone except along the midline sinus which is particularly sticky to the bone.
  c. Grab the bone with the forceps and slowly slide it to the side for its removal (Figure 4B).
  d. Stop bleeding by placing gelatin sponges on the bleeding sites.

***Note:*** The bone should be detached from the sinus by sliding laterally. The sinus will be damaged if the bone is pulled upward.

***Note:*** The frontal bones are stickier to the dura than the parietal bone. If the bones do not easily detach from the dura, carefully slide in the fine forceps between the bone and the dura.

***Note:*** We leave the dura intact to minimize inflammation.

22. Clean the glass plug with saline and Kimwipe.
23. Place the glass plug on the exposed cortex.
24. Push the glass plug down using the micromanipulator (Figure 4D). We use a sharpened wooden bar that is attached to the manipulator.

***Note:*** We do not need to push the full ∼440 μm height of the protrusion glass into the cranial window in most cases unless the skull is very thick. However, adding gentle pressure to the brain at this step is critical for stable two-photon calcium imaging from the cortical areas near the midline sinus of behaving mice. If the pressure is too weak, the midline sinus dilates and pushes the medial cortical areas such as the retrosplenial cortex when mice move during the behavior task. However, the pressure should not be so much that it blocks the blood flow in the sinus. The pressure should be adjusted based on the blood flow. We did not observe obvious effects on neural activity from this procedure.

25. Remove extra saline between the base glass and the skull using Kimwipe.
26. Fill the gap between the base glass and the skull with 3M Vetbond using a syringe, and wait for at least 30 s.
27. Remove extra 3M Vetbond using Kimwipe.
28. Put Krazy glue (cyanoacrylate glue) around the edges of the window using a syringe with a 25G needle.
29. Lift the wooden bar after making sure that the glass plug is stably glued to the skull (Figure 4E). It should be stable even before the Krazy glue dries.

### Fixing the head bar and the cranial window

**Timing: 40 min**

This section describes the steps to fixate the head bar and the cranial window using Krazy glue and dental acrylic cement. Use a syringe with 25G needle for Krazy glue, and use double-ended carver to mix and apply cement.

**Figure 5:**
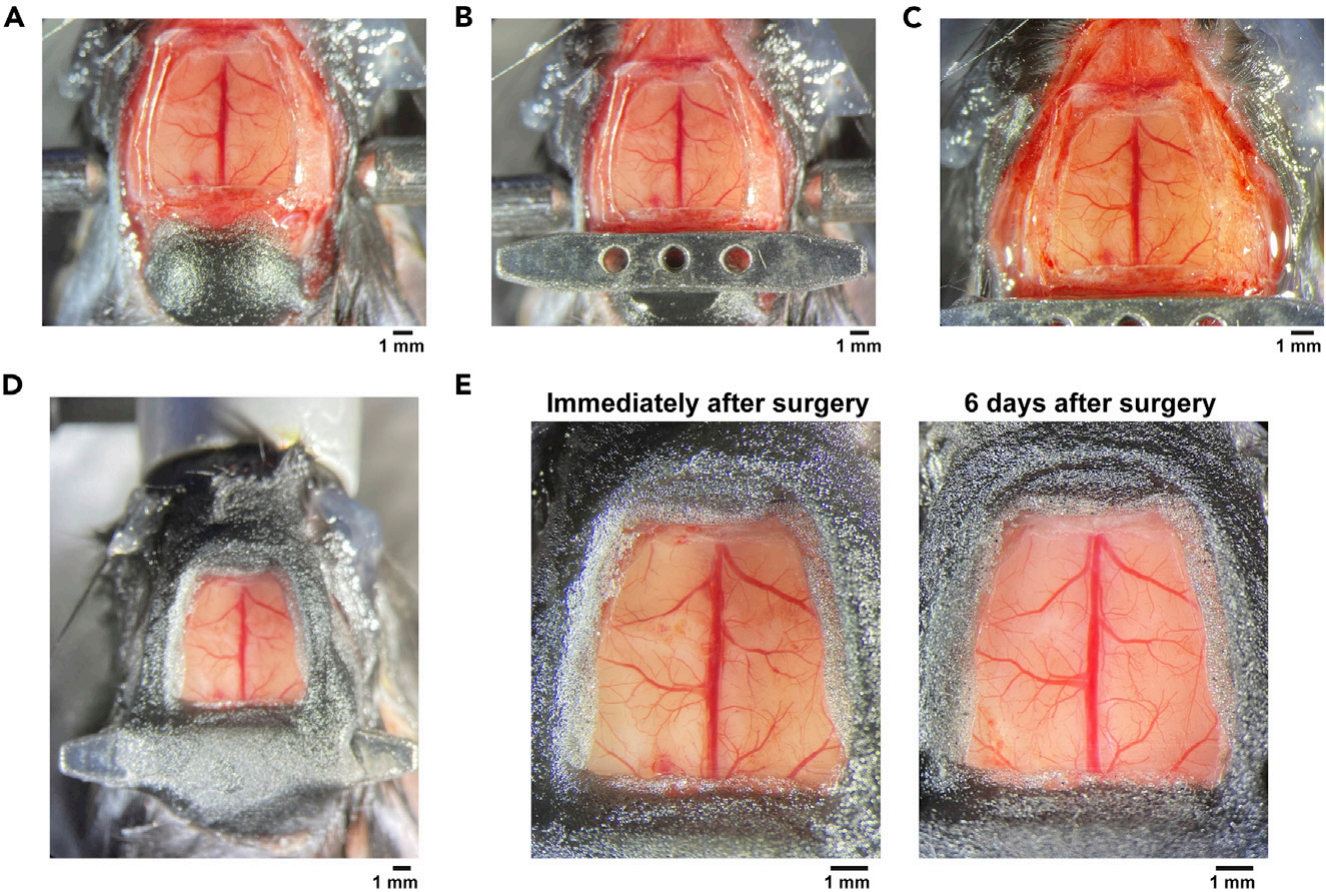
Fixing the head bar and the cranial window (A) Dental cement is mounted on the back muscle until it reaches the height of the cerebellum skull. (B) Head bar is glued on the cement and skull. (C) All exposed skull surface is covered by Krazy glue. (D) Dental cement is mounted on the skull to make a wall. (E) Cranial windows immediately after the surgery (left) and 6 days after the surgery (right). Note that the residual bleeding immediately after the surgery has cleared.
31. Place the head bar behind the glass window (Figure 5B).
32. After making sure that the head bar is roughly parallel to the skull, apply Krazy glue to secure the position of the head bar.
33. Cover all exposed skull with Krazy glue, including the lateral areas that were exposed in step 9 (Figure 5C).
34. Apply dental cement using double-ended carver.
35. Make a wall around the glass window by repeatedly applying the Krazy glue and the dental cement (Figure 5D).

***Note:*** The head bar also needs to be covered to achieve sufficient stability during imaging.

***Note:*** The wall height is matched to the height of the head bar in our preparation, but it can be adjusted if working distance of the objective lens is the limiting factor.

***Note:*** The first layer on the skull surface and the head bar should be always the Krazy glue instead of the dental cement because the dental acrylic cement does not strongly stick to the skull.

30. Cover the exposed muscles behind the cerebellum with a thin layer of Krazy glue and mount dental acrylic cement until the height becomes roughly equal to the skull above the cerebellum (Figure 5A).

36. Inject buprenorphine (0.1 mg/kg) subcutaneously.
37. After the cement dries, clean the glass window with a cotton swab soaked in 70% ethanol and take a photo of the cranial window (Figure 5E). This photo and another photo from step 15 will be used to find the target cortical areas during two-photon imaging.
38. Transfer the mouse to an animal cage and leave the cage on a heating pad until the mouse recovers from anesthesia. If water-restriction is necessary for the animal training, give at least 5 days of recovery after surgery before starting the water-restriction.

### Perform two-photon calcium imaging from the target cortical areas

**Timing: 15 min (excluding the imaging and the image registration time)**

This section describes the steps to find the target cortical areas and perform two-photon calcium imaging.

**Figure 6:**
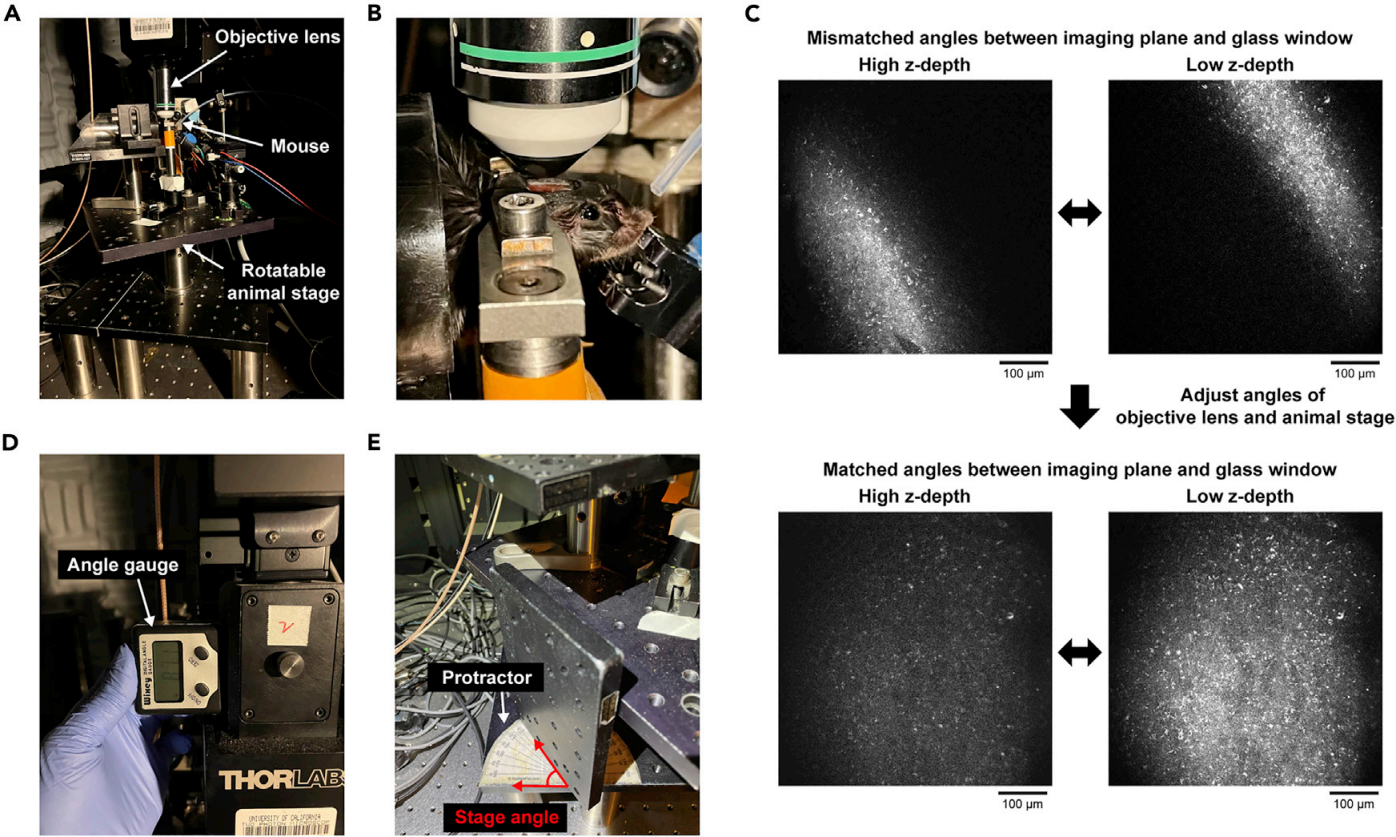
Preparation for two-photon imaging (A) Head-fixation stage under a two-photon microscope. The animal stage is rotatable. (B) High zoom image of a head-fixed mouse. (C) Angular discrepancy between the focal plane and the glass window can be checked by the reflected light on the glass window. Light will be reflected along a line when there is angular mismatch, while the light will be uniformly reflected on the glass when the angles are matched. Adjust the angles of both the animal stage and the objective lens to achieve uniform light reflection on the glass window. (D) Measure the rotational angle of the microscope (objective lens) using an angle gauge for reproducibility. (E) Measure the horizontal angle of the animal stage for reproducibility.

***Note:*** If the mouse is not pre-trained under head-fixation, we recommend habituating the mouse to the head-fixed condition at least for a few days before collecting calcium imaging data.

39. Find the cortical vasculature patterns within the target field-of-views (FOVs).
  a. Check the vasculature patterns within the target FOVs on the photo from the step 15 (Coordinate photo).
  b. Find the corresponding vasculature patterns on the photo from the step 37 (Post-surgery photo).
40. Head-fix the mouse under the two-photon microscope (Figures 6A and 6B).

41. Perform two-photon imaging with a water-immersion objective lens.
42. Find the vasculature patterns of the target FOVs by referencing the photos from the step 39.
43. Adjust the angles of the animal stage and the objective lens to make the glass window perpendicular to the light path through the objective lens.
  a. To find the angular discrepancy between the glass window and the objective lens, image the glass window while repeatedly moving the z-depth of the focal plane up and down around the window.
  b. The excitation light will be reflected along a line when there is an angular discrepancy, and the moving direction of the line during the movement of z-depth tells the directions to which the angles of the animal stage and the objective lens need to be corrected (Figure 6C).
  c. After correcting the angular discrepancy, the excitation light will be uniformly reflected on the glass window.
44. Move the objective lens down to the target depth.
45. Start the calcium imaging (e.g., ScanImage for data acquisition) and the behavior task.
46. After imaging, record the angles of the animal stage and the objective lens (Figures 6D and 6E).
47. Remove the motion artifacts and distortions from saved images using image registration algorithms such as PatchWarp (Hattori and Komiyama, 2022b).

### Track the identical neural population across sessions for longitudinal analyses

**Timing: 15 min (excluding the imaging time)**

This section describes the steps to find the identical neural population between different imaging sessions for longitudinal analyses.

48. Create a reference image of each target FOV by averaging all frames of the registered images from a reference session. PatchWarp automatically generates the reference image at the step 47.
49. Set the angles of the animal stage and the objective lens to the recorded angles from the step 46.
50. Find the target FOV based on the cortical vasculature patterns and move the focus down to the same depth as before.
51. Compare the current FOV with the FOV in the reference image, and adjust the imaging depth and the animal stage angle.

***Note:*** Vasculatures are more reliable landmarks than neurons for the FOV matching, because only neurons that are currently active are often visible with GCaMP signals.

***Note:*** The FOVs do not need to match completely because the FOVs from different imaging sessions can be registered post-hoc (e.g., Across-session registration function of PatchWarp).

52. Start the calcium imaging and the behavior task.

## Expected outcomes

**Figure 7:**
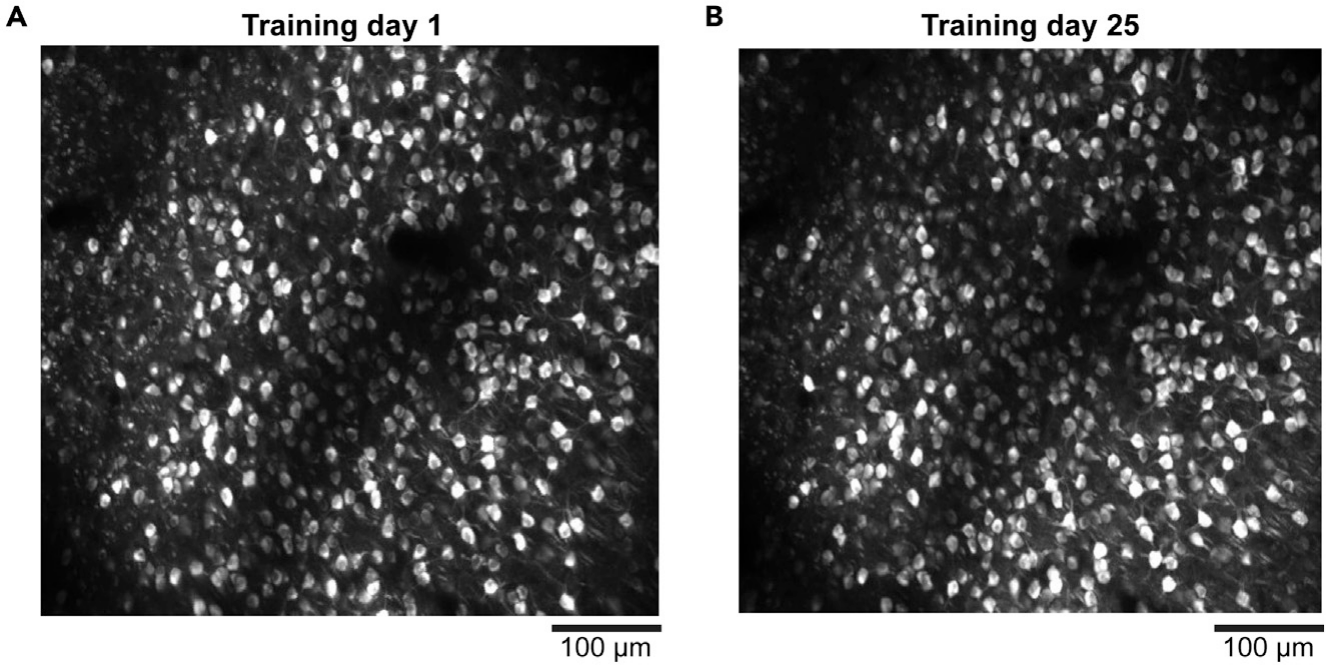
Longitudinal imaging of identical neural population (A and B) Example images from the retrosplenial cortex on day 1 (A) and day 25 (B) of the training in a value-based decision task. Max-intensity projection of all frames for each session. Images were denoised by temporal moving averaging (window size of 50 frames) before the max-intensity projections.

Standard craniotomy exposes only a small portion of the dorsal cortex (Augustinaite and Kuhn, 2020; Goldey et al., 2014; Holtmaat et al., 2009; Oomoto et al., 2021), which limits the amount of data that can be collected from individual mice. In contrast, our cranial window covers most dorsal cortical areas in individual mice. The large window saves a lot of experimental time and cost because we do not need to prepare separate animals for different cortical areas. This point is particularly significant when the animal training takes a long time. Furthermore, we can compare the neural activity between different cortical areas within each animal. Using this protocol, we performed longitudinal two-photon calcium imaging from 6 distant dorsal cortical areas for over a month during training in a value-based decision task (Hattori and Komiyama, 2022a; Hattori et al., 2019). Example images of a longitudinally tracked neural population from the retrosplenial cortex are shown (Figures 7A and 7B).

## Limitations

The lateral dimension of the cranial window with this protocol is limited by the curvature of the cortex. If more lateral areas need to be included in the cranial window, curve the glass window (Kim et al., 2016) or use curved transparent polymer (Ghanbari et al., 2019). If cellular resolution is not necessary for calcium imaging, a skull-intact surgical preparation is sufficient for one-photon wide-field calcium imaging (Ren and Komiyama, 2021).

## Troubleshooting

### Problem 1

Glass plug does not fit the cranial window (step 23).

### Potential solution

Try to make the cranial window larger by removing remaining thinned skulls around the edges of the window using fine forceps. If the glass plug still does not fit, cover the exposed cortex with gelatin sponge soaked in saline and make a new glass plug with adjusted size. We recommend to prepare multiple glass plugs with slightly different sizes in steps 2–5 so that the best size can be selected in step 23.

### Problem 2

Bleeding does not stop with gelatin sponges during surgery (steps 18–21).

### Potential solution

If gelatin sponges alone do not stop bleeding, gently push the gelatin sponge against the bleeding site with dry Kimwipe. After the bleeding stops, soak the sponge with saline and slowly remove the sponge.

### Problem 3

Bone growth covered the cranial window.

### Potential solution

Some areas may be covered by bone growth after surgery. If a part of the cranial window was covered by a thin layer of bone, image only the remaining areas in the window. The chance of bone growth can be minimized by sufficiently pushing in the glass plug at the step 24.

### Problem 4

Cranial window became cloudy (white color).

### Potential solution

It may be due to infection or inflammation. Inject additional baytril (10 mg/kg) and dexamethasone (2 mg/kg) subcutaneously daily or every other day.

### Problem 5

The head bar and cement often come off during animal training or imaging (steps 39–52).

### Potential solution

The cement was probably loose on the skull. Make sure to remove all connective tissue on the skull surface (step 10), and cover all exposed skull with Krazy glue (step 33).

## Resource availability

### Lead contact

Further information and requests for resources and reagents should be directed to and will be fulfilled by the lead contact, Takaki Komiyama (tkomiyama@ucsd.edu).

### Materials availability

This study did not generate new materials.

## Data Availability

Data are available from the corresponding author upon reasonable request. PatchWarp code for image registrations is available at GitHub (https://github.com/ryhattori/PatchWarp).
